# The clinical application value of 3.0T magnetic resonance T2 mapping imaging in evaluating the degree of acetabular cartilage degeneration in joint replacement surgery running title: MRI and acetabular cartilage degeneration

**DOI:** 10.1186/s13018-024-04898-3

**Published:** 2024-07-19

**Authors:** Xiang Peng, An-min Xie, Hua-gang Fan, Hong-liang Zhu, Di Yang, De-en Wan, Fei He, Chong Luo, Hao Li, Feng Shuang

**Affiliations:** 1 Department of Orthopedics, The 908th Hospital of Chinese People’s Liberation Army Joint Logistics Support Force, No.1028 Jinggangshan Street, Qingyunpu District, Nanchang, 330002 Jiangxi Province China; 2 Department of Diagnostic Radiology, The 908th Hospital of Chinese People’s Liberation Army Joint Logistics Support Force, Nanchang, 330002 Jiangxi Province China; 3 Department of Quality Management, The 908th Hospital of Chinese People’s Liberation Army Joint Logistics Support Force, Nanchang, 330002 Jiangxi Province China

**Keywords:** Magnetic resonance imaging, T2 mapping, Acetabular cartilage degeneration, Femoral neck fracture

## Abstract

**Background:**

To explore and compare the values of 3.0T magnetic resonance imaging (MRI) T2 mapping in evaluating the degree of acetabular cartilage degeneration in hip replacement surgery.

**Methods:**

A total of 26 elderly patients with femoral neck fractures who were scanned in 3.0T MRI T2 mapping quantification technique were included. Basing on MRI images, the degree of acetabular cartilage degeneration was classified into Grade 0, 1, 2, 3 and 4, according to the International Cartilage Repair Society (ICRS) scores. In addition, 8 healthy volunteers were included for control group.

**Results:**

By comparison with health population, T2 relaxation values in the anterior, superior, and posterior regions of acetabular cartilage in patients with femoral neck fracture were obviously increased (*P* < 0.001). Among the patients with femoral neck fractures, there were 16 hip joint with Grade 1–2 (mild degeneration subgroup) and 10 hip joints with Grade 3–4 (severe degeneration subgroup), accounting for 61.54% and 38.46%, respectively. Additionally, T2 relaxation values in the anterior and superior bands of articular cartilage were positively related to the MRI-based grading (*P* < 0.05); while there was no significant difference of T2 relaxation values in the posterior areas of articular cartilage between severe degeneration subgroup and mild degeneration subgroup (*P* > 0.05). Importantly, acetabular cartilage degeneration can be detected through signal changes of T2 mapping pseudo-color images.

**Conclusion:**

3.0T MRI T2 mapping technology can be used to determine the degree of acetabular cartilage degeneration, which can effectively monitor the disease course.

## Background

As the global population ages, the incidence of hip fracture in elderly patients has increased over the past three decades, suggesting that hip fracture remains a universal public health challenge [1]. Femoral neck fracture is a special type of hip fracture, and most elderly patients with femoral neck fracture have the abnormalities of acetabular cartilage, which may impact postoperative outcomes such as pain and function [2]. To evaluate the degree of cartilage degeneration, X-ray, computed tomography (CT), and magnetic resonance imaging (MRI) were commonly used in clinical diagnosis [3, 4]. However, conventional X-ray, as a currently economical and readily available imaging technique, can only indirectly determine the cartilage degeneration by observing the degree of joint space narrowing [5]. The introduction of MRI has largely replaced hip arthroscopy as an invasive examination for direct analysis of cartilage [6]. Conventional MRI can evaluate the morphological changes of inarticular cartilage; but, it is still difficult to display the structure of articular cartilage [7]. For quantification of cartilage composition, MRI mapping techniques can support disease characterization, disease progression monitoring, and therapy response [8].

Recently, quantitative MRI techniques, such as T1, T2, and T2^*^ mapping, have been shown to evaluate the cartilage degeneration of hip joint and shoulder joint [9–11]. Among them, T2 mapping is sensitive to the changes in the contents of water and collagen fiber, and cartilage orientation [12]. Especially in hip, high resolution (3.0 T or above) is needed for adequate quantitative imaging, mainly as a result of the thinner cartilage layer, and close contact between the acetabulum and femoral cartilage [13].

Based on the unique advantages of rapid imaging, high image resolution, easy operation and three-dimensional quantitative analysis of cartilage, T2 mapping technology can provide a reliable basis for judging the degree of acetabular cartilage degeneration. In the current study, 3.0T MRI T2 mapping quantification technique was used to assess the acetabular cartilage degeneration in elderly patients with femoral neck fracture, aiming to provide support for clinical diagnosis and treatment decisions.

## Methods

### Study population

The study was retrospectively included the patients with femoral neck fracture who underwent hip replacement surgery in Orthopedic Joint Disease Area of the 908th Hospital of Joint Logistic Support Force from January 2023 to June 2023. A total of 26 elderly patients were collected, including 12 males and 16 females, with an average age of 77.1 ± 8.5 years. Inclusion criteria were patients who were (1) diagnosed as femoral neck fracture by imaging; (2) prepared for hip replacement surgery; (3) tolerance to MRI examination; (4) aged ≥ 60 years old.

In addition, 8 healthy volunteers (4 males and 4 females, aged 18–25 years, 21.2 ± 2.6 years) were included. The inclusion criteria for healthy volunteers were (1) with normal BMI; (2) no discomfort symptoms such as hip pain or limited mobility; (3) signed the informed consent form. Exclusion criteria were as follows: (1) pregnant or lactating population; (2) population with MRI related contraindications (such as pacemaker implantation or claustrophobia). This study was approved by the Medical Ethics Committee of the 908th Hospital of Chinese People’s Liberation Army Joint Logistics Support Force (908YYLL103). All participants have signed the informed consent form.

### MRI examination

The 3.0T MRI (a United Imaging MR770 scanner) with 12-channel body coil was performed to scan using conventional sagittal FSE T1WI, sagittal FRFSE T2WI/PDWI and coronal FRFSE T2WI sequences in all patients. The 5-echo axial FSE sequences were scanned for T2-mapping imaging and the scanning parameters were as follows: TR = 861.5 ms, TE = 6.71/15.65/24.6/33.54/42.48 ms, thick layer of 4.0 mm, layer spacing of 0.6 mm, FOV = 18 cm×18 cm, 3680 × 100 matrix, NEX = 1, and the scanning time of 3.09 min.

### Image processing

Based on MRI images, acetabular cartilage degeneration was graded, according to International Cartilage Repair Society (ICRS) score [14]. Specific grading, where “Grade 0” was defined as normal cartilage; “Grade 1” was defined as local signal enhancement of articular cartilage, without cartilage defect; “Grade 2” was defined as erosion less than 50% of cartilage thickness; “Grade 3” was defined as defect greater than 50% of articular cartilage depth, with or without small bone ulcers; “Grade 4” was defined as full-layer articular cartilage defect with bone dissection. Grade 1 to 2 was classified as mild degeneration subgroup and Grade 3 to 4 as severe degeneration subgroup. Two or more attending physicians read the images separately, and if the grading was inconsistent, the two physicians need to discuss before making decision.

**Fig. 1:**
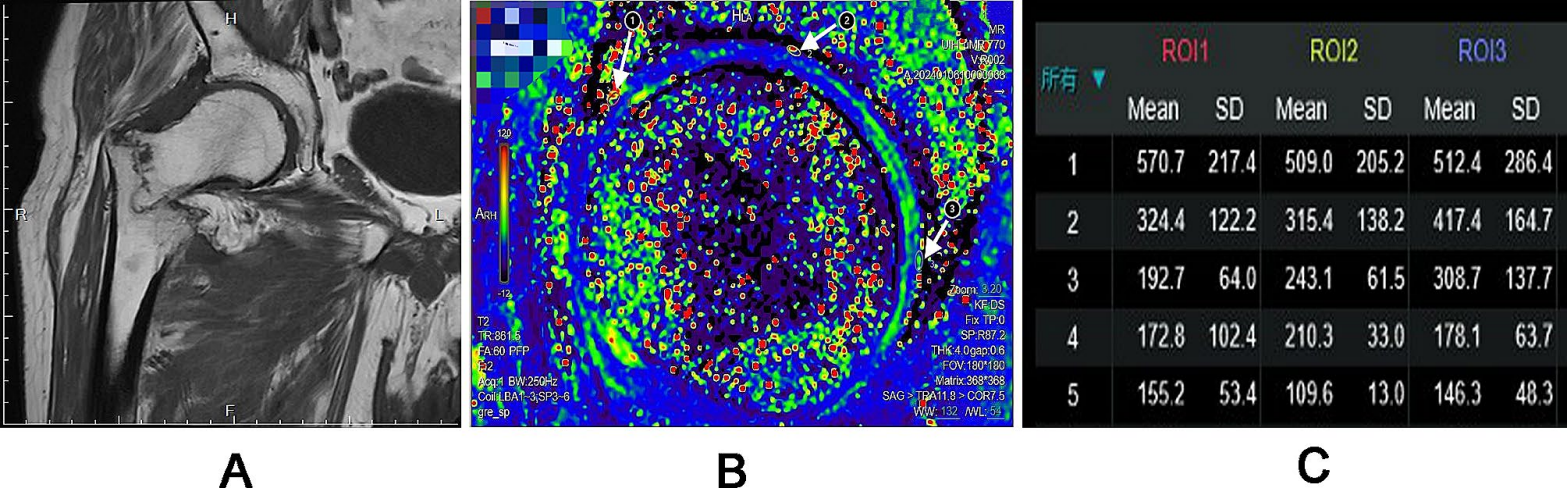
A typical elderly patient with right femoral neck fracture **(A)**, and T2-mapping pseudo-color images demonstrated the anterior ROI (ROI1), superior ROI (ROI2) and posterior ROI (ROI3) **(B)** and corresponding information about the number of pixels in each ROIs **(C)**. Of note, because of low cartilage thickness in cases with cartilage degeneration, so, during manual selection on ROIs of the acetabular cartilage, the images can be enlarged to avoid the femoral cartilage and subacetabular bone as much as possible. ROI, region of interest

MRI datasets were transferred to a workstation, and pseudo-color T2 mapping images was generated by the Functool software package. Using the sagittal PDWI image as reference, the region of interest (ROI) on the pseudo-color images was manually drawn in acetabular cartilage. Of note, during the measurement, images were enlarged to avoid the femoral cartilage and subacetabular bone as much as possible. At the same time, a median sagittal slice was taken at the apex of femoral head (that is, when the concentric circle diameter is maximum) to ensure the measurement in same area. The results of pseudo-color mapping in elderly patients were shown in Fig. 1A-C, respectively. For reliability assessment, T2 relaxation values in the anterior, superior, and posterior areas of acetabulum were measured at least 3 times in each place and then averaged.

### Statistical analysis

All data were statistically analyzed using SPSS 20.0 software. T2 relaxation values of acetabular cartilage were expressed as mean ± standard deviation (SD). The comparison of T2 relaxation values between the two groups was performed by student’s *t* test, and *P* < 0.05 was considered statistically significant.

## Results

### Comparison of T2 relaxation value in patients with femoral neck fracture and healthy population

**Fig. 2:**
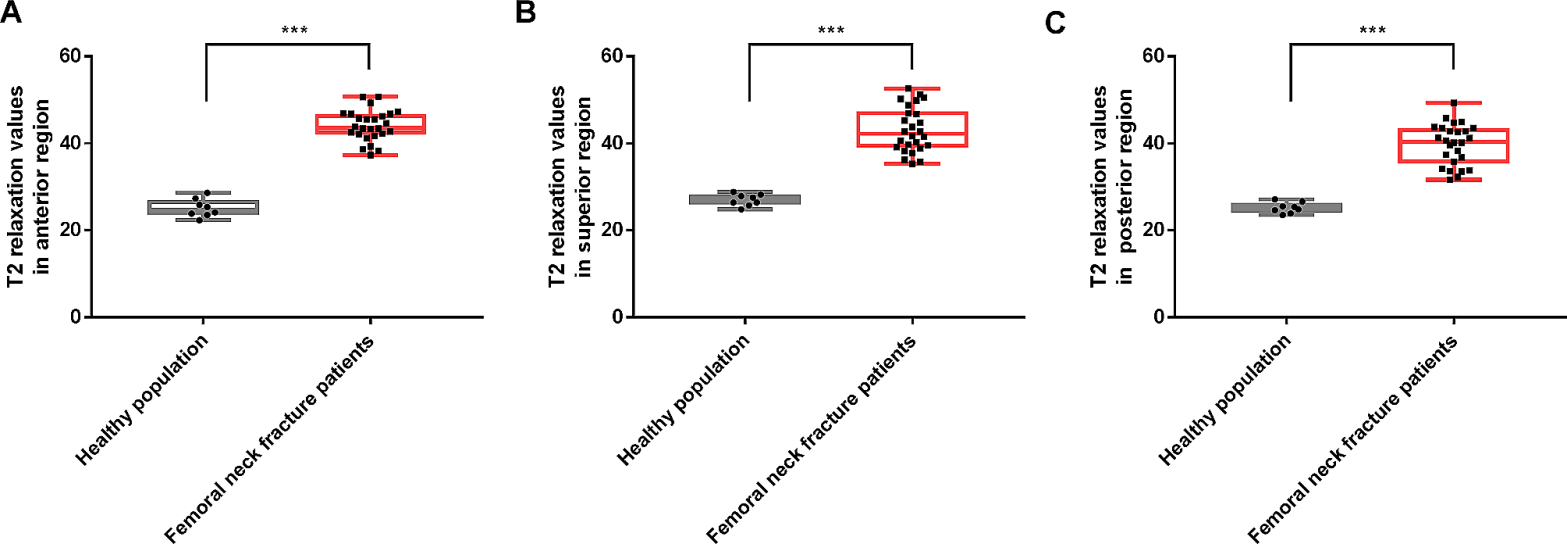
Individual T2 relaxation values in anterior **(A)**, superior **(B)** and posterior **(C)** regions of acetabular cartilage in patients and healthy population. ^***^P < 0.001

As displayed in Tables 1**and** Fig. 2, T2 relaxation values in the anterior, superior, and posterior regions of acetabular cartilage in patients with femoral neck fracture were obviously increased (*P* < 0.001).

**Table 1:** Comparison of T2 relaxation values in patients with femoral neck fracture and healthy population

Groups	Cases	Anterior acetabular cartilage	Superior acetabular cartilage	Posterior acetabular cartilage
Femoral neck fracture patients	26	44.06 ± 3.56	43.09 ± 5.17	39.76 ± 4.71
Healthy population	8	25.11 ± 2.10	26.96 ± 1.36	25.17 ± 1.24
*t* value		14.20	8.63	8.59
*p* value		< 0.001	< 0.001	< 0.001

### Comparison of T2 relaxation value in femoral neck fracture patients with mild degeneration and severe degeneration subgroup

As displayed in Table 2, T2 relaxation values of anterior and superior articular cartilage in the severe degeneration subgroup were statistically higher than those in the mild degeneration subgroup (*P* < 0.05), however there was no statistical significance in the T2 relaxation values of posterior articular cartilage between the severe degeneration subgroup and mild degeneration subgroup (*P* > 0.05).

**Table 2:** Comparison of T2 relaxation values in femoral neck fracture patients with mild degeneration and severe degeneration subgroup

Groups	Cases	Anterior acetabular cartilage	Superior acetabular cartilage	Posterior acetabular cartilage
Mild degeneration subgroup	16	42.58 ± 2.94	41.49 ± 3.91	38.97 ± 4.35
Severe degeneration subgroup	10	46.43 ± 3.29	45.65 ± 6.08	41.03 ± 5.21
*t* value		-3.10	-2.13	-1.09
*p* value		0.005	0.043	0.286

### Comparison of T2 mapping pseudo-color images in patients with femoral neck fracture and healthy population

**Fig. 3:**
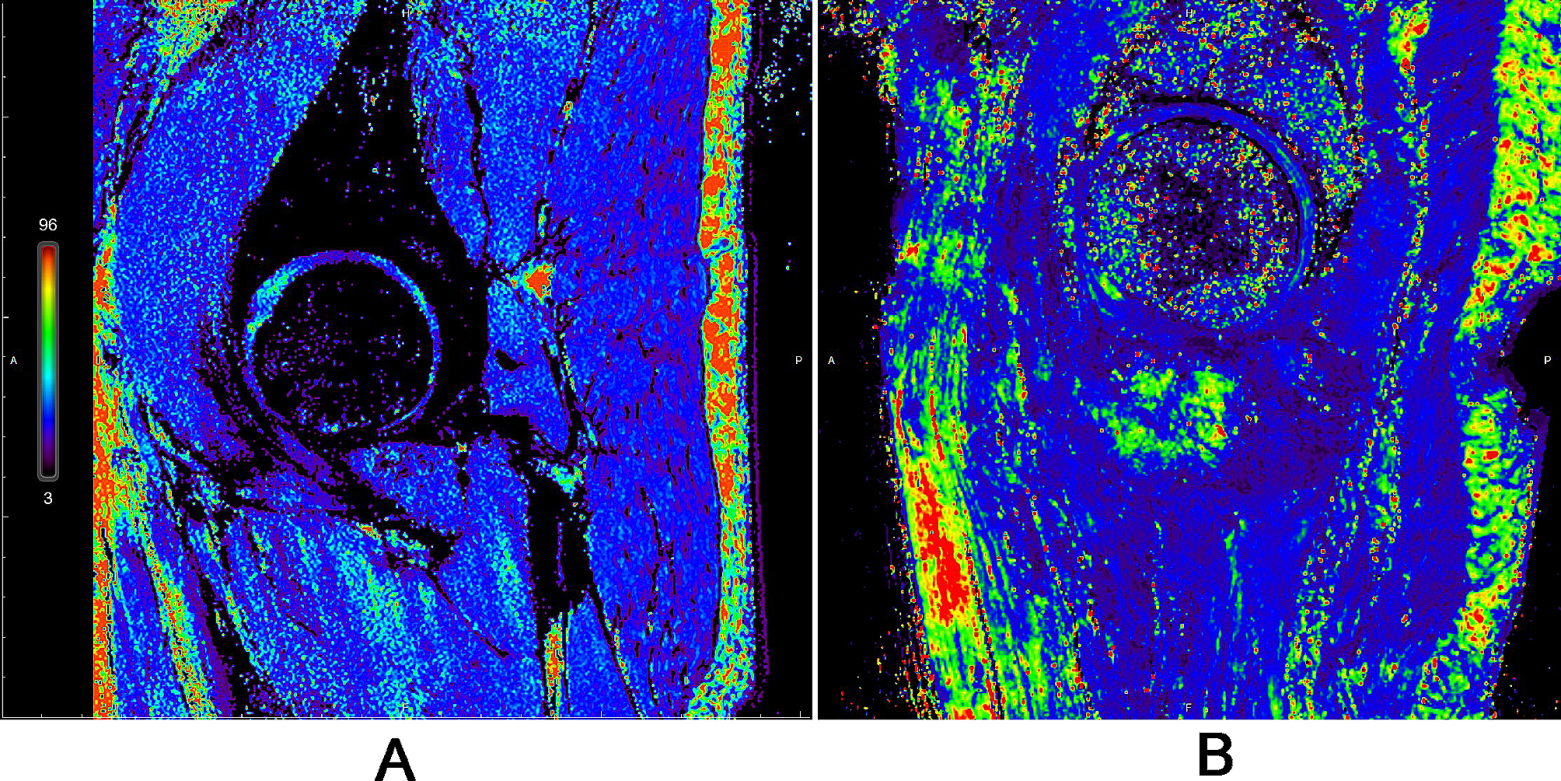
T2 mapping pseudo-color images in patients with femoral neck fracture and healthy population. **(A)** Representative images of healthy population (Male, 20 years old), **(B)** T2 mapping pseudo-color images of patients with femoral neck fracture (female, 90 years old)

T2-mapping pseudo-color images showed that the cartilage morphology of healthy population was complete and continuous with uniform color levels. In patients with femoral neck fracture, the cartilage morphology was interrupted and color scales were mixed (Fig. 3).

## Discussion

T2 mapping is as an objective and non-invasive method for assessing acetabular cartilage injury and can provide information comparable to arthroscopy [15]. As for T2 relaxation value in acetabular cartilage, the elevation is due to the decrease of proteoglycan content and increases of water content and water mobility associated with disruption of collagen networks. In specific, Nieminen et al. [16] have observed an increase of T2 relaxation value in the superficial area of bovine cartilage after degradation of collagen structure by enzyme treatment. Another study has proved that T2 relaxation rate is closely correlated with the water content of human knee joint cartilage [17]. Additionally, Wayne et al. [18] have showed that T2 relaxation value is significantly negatively correlated with proteoglycan content or cartilage hardness. Clinically, T2 relaxation value is significantly positively correlated with disease severity, as determined by higher T2 relaxation value in patients with femoral head necrosis compared with healthy controls [19]. In line, our study uncovered that T2 relaxation values in the anterior, superior, and posterior regions of acetabular cartilage in femoral neck fracture patients were notably increased by comparison with healthy population, supported the roles of T2 relaxation values on evaluating cartilage degeneration.

Meanwhile, our study also displayed that T2 relaxation values of anterior and superior regions of acetabular cartilage in the severe degeneration subgroup were higher than those of the mild degeneration subgroup; while the T2 relaxation values of posterior cartilage between the two groups was not statistically significant. Aforementioned findings was consistent with prior study [20]. Importantly, our study also showed that the posterior cartilage seems to develop degenerative changes later than the anterior and superior parts. In line, Teichtahl et al. [21] have stated that almost acetabular cartilage defects are located in anterior and superior areas of acetabulum. Moreover, Wong et al. [22] have also found that the T2 relaxation value in the anterior region of hip joint is significantly higher than that in the posterior region, and tends to be higher than that in the superior region. The possible reason is that contact stress in anterior and superior regions is more concentrated; on the other hand, the anterior region of acetabular cartilage has less blood supply than other regions, and the self-repair ability is relatively poor. Therefore, measuring T2 values in anterior and superior parts of acetabulum may have greater clinical significance.

The shortcomings of this study were as follows: first, at present, T2 mapping studies on hip cartilage mainly focus on hip osteoarthritis, hip dysplasia, hip impingency syndrome and other fields, and there are few literatures and reports on its application in hip replacement. Second, the limited number of cases included in this study may lead to results bias; third, ROIs selection and pseudo-color signal judgment are subjective to a certain extent, which may affect the accuracy of the results.

## Conclusions

3.0T MRI T2 mapping technology can be used to determine the degree of acetabular cartilage degeneration based on T2 value and pseudo-color images, which provides the support for clinical diagnosis and treatment decisions.

## Data Availability

All data generated or analysed during this study are included in this published article.

## References

[1] Feng JN, Zhang CG, Li BH, Zhan SY, Wang SF, Song CL. Global burden of hip fracture: The Global Burden of Disease Study. Osteoporos Int. 2024;35(1):41–52. 37704919 10.1007/s00198-023-06907-3

[2] Ochi H, Kobayashi H, Baba T, Nakajima R, Kurita Y, Kato S et al. Acetabular cartilage abnormalities in elderly patients with femoral neck fractures. SICOT-J. 2022;8. 10.1051/sicotj/2022022 PMC9196023 35699460

[3] Thevenot J, Hirvasniemi J, Pulkkinen P, Määttä M, Korpelainen R, Saarakkala S, et al. Assessment of risk of femoral neck fracture with radiographic texture parameters: a retrospective study. Radiology. 2014;272(1):184–91. 24620912 10.1148/radiol.14131390

[4] Nelson BB, Kawcak CE, Barrett MF, McIlwraith CW, Grinstaff MW, Goodrich LR. Recent advances in articular cartilage evaluation using computed tomography and magnetic resonance imaging. Equine Vet J. 2018;50(5):564–79. 29344988 10.1111/evj.12808

[5] Vignon E, Conrozier T, Piperno M, Richard S, Carrillon Y, Fantino O. Radiographic assessment of hip and knee osteoarthritis. Recommendations: recommended guidelines. Osteoarthritis Cartilage. 1999;7(4):434–6. 10419791 10.1053/joca.1999.0235

[6] Keeney JA, Peelle MW, Jackson J, Rubin D, Maloney WJ, Clohisy JC. Magnetic resonance arthrography versus arthroscopy in the evaluation of articular hip pathology. Clin Orthop Relat Res. 2004(429):163–9. 10.1097/01.blo.0000150125.34906.7d 15577482

[7] Jazrawi LM, Alaia MJ, Chang G, Fitzgerald EF, Recht MP. Advances in magnetic resonance imaging of articular cartilage. J Am Acad Orthop Surg. 2011;19(7):420–9. 21724921 10.5435/00124635-201107000-00005

[8] Matzat SJ, van Tiel J, Gold GE, Oei EH. Quantitative MRI techniques of cartilage composition. Quant Imaging Med Surg. 2013;3(3):162–74. 23833729 10.3978/j.issn.2223-4292.2013.06.04 PMC3701096

[9] Cloos MA, Assländer J, Abbas B, Fishbaugh J, Babb JS, Gerig G, et al. Rapid Radial T(1) and T(2) mapping of the hip articular cartilage with magnetic resonance fingerprinting. J Magn Reson Imaging. 2019;50(3):810–5. 30584691 10.1002/jmri.26615 PMC6591100

[10] Cao G, Gao S, Xiong B. Application of quantitative T1, T2 and T2* mapping magnetic resonance imaging in cartilage degeneration of the shoulder joint. Sci Rep. 2023;13(1):4558. 36941288 10.1038/s41598-023-31644-2 PMC10027866

[11] Lazik A, Theysohn JM, Geis C, Johst S, Ladd ME, Quick HH, et al. 7 Tesla quantitative hip MRI: T1, T2 and T2* mapping of hip cartilage in healthy volunteers. Eur Radiol. 2016;26(5):1245–53. 26314482 10.1007/s00330-015-3964-0

[12] Leskinen HPP, Hänninen NE, Nissi MJ. T(2)orientation anisotropy mapping of articular cartilage using qMRI. Phys Med Biol. 2023;68(8). 10.1088/1361-6560/acc169 36867883

[13] Nishii T, Tanaka H, Sugano N, Sakai T, Hananouchi T, Yoshikawa H. Evaluation of cartilage matrix disorders by T2 relaxation time in patients with hip dysplasia. Osteoarthritis Cartilage. 2008;16(2):227–33. 17644363 10.1016/j.joca.2007.06.003

[14] Paatela T, Vasara A, Nurmi H, Kautiainen H, Kiviranta I. Assessment of cartilage repair quality with the International Cartilage Repair Society score and the Oswestry Arthroscopy score. J Orthop Research^®^. 2020;38(3):555–62. 31608499 10.1002/jor.24490

[15] Wuennemann F, Kintzelé L, Braun A, Zeifang F, Maier MW, Burkholder I, et al. 3-T T2 mapping magnetic resonance imaging for biochemical assessment of normal and damaged glenoid cartilage: a prospective arthroscopy-controlled study. Sci Rep. 2020;10(1):14396. 32873848 10.1038/s41598-020-71311-4 PMC7462998

[16] Nieminen MT, Töyräs J, Rieppo J, Hakumäki JM, Silvennoinen J, Helminen HJ, et al. Quantitative MR microscopy of enzymatically degraded articular cartilage. Magn Reson Med. 2000;43(5):676–81. 10800032 10.1002/(SICI)1522-2594(200005)43:5<676::AID-MRM9>3.0.CO;2-X

[17] Lüsse S, Claassen H, Gehrke T, Hassenpflug J, Schünke M, Heller M, et al. Evaluation of water content by spatially resolved transverse relaxation times of human articular cartilage. Magn Reson Imaging. 2000;18(4):423–30. 10788720 10.1016/S0730-725X(99)00144-7

[18] Wayne JS, Kraft KA, Shields KJ, Yin C, Owen JR, Disler DG. MR imaging of normal and matrix-depleted cartilage: correlation with biomechanical function and biochemical composition. Radiology. 2003;228(2):493–9. 12893905 10.1148/radiol.2282012012

[19] Han X, Hong G, Chen L, Zhao M, Guo Y, Xu L, et al. T(1) ρ and T(2) mapping for the determination of articular cartilage denaturalization with osteonecrosis of the femoral head: a prospective controlled trial. J Magn Reson Imaging. 2019;49(3):760–7. 30461119 10.1002/jmri.26267

[20] Meng XW. The value of joint oblique scan and T2 mapping in the diagnosis of hip joint cartilage injury. Jilin University; 2021.

[21] Teichtahl AJ, Wang Y, Smith S, Wluka AE, Giles GG, Bennell KL, et al. Structural changes of hip osteoarthritis using magnetic resonance imaging. Arthritis Res Ther. 2014;16(5):466. 25304036 10.1186/s13075-014-0466-4 PMC4212104

[22] Wong TT, Quarterman P, Lynch TS, Rasiej MJ, Jaramillo D, Jambawalikar SR. Feasibility of ultrashort echo time (UTE) T2* cartilage mapping in the hip: a pilot study. Acta Radiol. 2022;63(6):760–6. 33926266 10.1177/02841851211011563

